# Ckb-Cryab-Drp1 axis enhances BMSC osteogenic differentiation and mouse alveolar bone healing through mitochondrial fission modulation

**DOI:** 10.1093/stcltm/szag056

**Published:** 2026-08-05

**Authors:** Mingxi Wang, Long Wang, Jingchan Wang, Shan Gao, Guoqing Li, Chunbo Tang

**Affiliations:** Department of Dental Implantology, The Affiliated Stomatological Hospital of Nanjing Medical University, Nanjing 210029, China; State Key Laboratory Cultivation Base of Research, Prevention and Treatment for Oral Diseases, Nanjing Medical University, Nanjing 210029, China; Jiangsu Province Engineering Research Center of Stomatological Translational Medicine, Nanjing Medical University, Nanjing 210029, China; Department of Dental Implantology, The Affiliated Stomatological Hospital of Nanjing Medical University, Nanjing 210029, China; State Key Laboratory Cultivation Base of Research, Prevention and Treatment for Oral Diseases, Nanjing Medical University, Nanjing 210029, China; Jiangsu Province Engineering Research Center of Stomatological Translational Medicine, Nanjing Medical University, Nanjing 210029, China; Department of Dental Implantology, The Affiliated Stomatological Hospital of Nanjing Medical University, Nanjing 210029, China; State Key Laboratory Cultivation Base of Research, Prevention and Treatment for Oral Diseases, Nanjing Medical University, Nanjing 210029, China; Jiangsu Province Engineering Research Center of Stomatological Translational Medicine, Nanjing Medical University, Nanjing 210029, China; Department of Dental Implantology, The Affiliated Stomatological Hospital of Nanjing Medical University, Nanjing 210029, China; State Key Laboratory Cultivation Base of Research, Prevention and Treatment for Oral Diseases, Nanjing Medical University, Nanjing 210029, China; Jiangsu Province Engineering Research Center of Stomatological Translational Medicine, Nanjing Medical University, Nanjing 210029, China; Department of Dental Implantology, The Affiliated Stomatological Hospital of Nanjing Medical University, Nanjing 210029, China; State Key Laboratory Cultivation Base of Research, Prevention and Treatment for Oral Diseases, Nanjing Medical University, Nanjing 210029, China; Jiangsu Province Engineering Research Center of Stomatological Translational Medicine, Nanjing Medical University, Nanjing 210029, China; Department of Dental Implantology, The Affiliated Stomatological Hospital of Nanjing Medical University, Nanjing 210029, China; State Key Laboratory Cultivation Base of Research, Prevention and Treatment for Oral Diseases, Nanjing Medical University, Nanjing 210029, China; Jiangsu Province Engineering Research Center of Stomatological Translational Medicine, Nanjing Medical University, Nanjing 210029, China

**Keywords:** Ckb-Cryab-Drp1 axis, BMSCs, osteogenic differentiation, alveolar bone healing, mitochondrial fission

## Abstract

Repairing bone defects resulting from trauma, infection, or tumor resection remains a significant challenge in clinical practice. Particularly in the oral and maxillofacial region, achieving predictable bone regeneration is especially difficult due to the complex anatomical structure and the critical need for optimal aesthetic and functional outcomes. A deeper understanding of the molecular mechanisms governing osteogenic differentiation is therefore essential to develop effective strategies for bone repair. Although creatine kinase B (Ckb) is known for its role in energy metabolism, its function in bone regeneration remains poorly understood. In this investigation, we found that Ckb is specifically expressed in jaw mesenchymal cells. Functional assays demonstrated that Ckb promoted osteogenic differentiation of bone marrow mesenchymal stem cells in vitro. Consistently, Ckb overexpression promoted alveolar bone healing in a mouse tooth-extraction model, while Ckb knockdown impaired bone regeneration. We discovered that Ckb localized to mitochondria and enhanced mitochondrial fission by increasing dynamin-related protein 1 (Drp1) Ser616 phosphorylation. The pro-osteogenic impact of Ckb was reduced by pharmacologically inhibiting mitochondrial fission with Mdivi-1. This result was replicated by Drp1 knockdown, indicating that Drp1 is essential for Ckb-mediated osteogenesis. Transcriptome sequencing identified **α**B-crystallin (Cryab) as a key downstream effector of Ckb, and co-immunoprecipitation confirmed a physical interaction between Ckb and Cryab. Crucially, Cryab overexpression rescued the osteogenic and mitochondrial dysfunction caused by Ckb knockdown. Collectively, our research reveals a unique Ckb-Cryab-Drp1 axis that controls mitochondrial dynamics to coordinate osteogenesis, offering a molecular basis for guiding MSC-based bone regeneration techniques.

## Graphical abstract

**Graphical Abstract:**
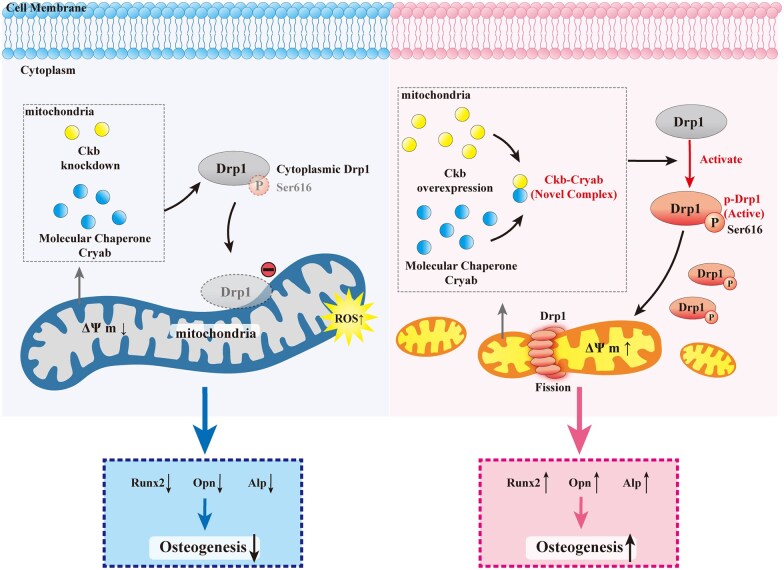

Significance statement This study identifies the creatine kinase B (Ckb)-Cryab-Drp1 axis as a novel regulator of osteogenesis via enhancing mitochondrial fission and function in bone marrow mesenchymal stem cells. These findings provide new insights into the molecular regulation of bone regeneration and expand the understanding of Ckb function from energy metabolism to mitochondrial dynamics.

## Introduction

Many oral disorders, such as periodontitis, trauma, tumors, and congenital deformities, can cause defects and loss of oral and maxillofacial bone tissue.^1^^,^^2^ These conditions can lead to multiple complications including masticatory dysfunction, aesthetic concerns, and speech impairments, significantly affecting patients’ quality of life.^3^ Additionally, maxillofacial bone abnormalities are a significant problem in prosthodontic rehabilitation and dental implantation. Although techniques such as autologous bone grafting and guided bone regeneration (GBR) are frequently employed in clinical practice,^3^^,^^4^ improving the effectiveness and caliber of bone regeneration is still a major objective in modern oral regenerative medicine. Bone marrow mesenchymal stem cells (BMSCs), owing to their self-renewal capacity and multi-lineage differentiation potential,^5^ are core seed cells in bone tissue engineering and regenerative medicine.^6^^,^^7^ BMSC differentiation into osteoblasts is a key component of the biological process of bone formation.^8^

Mitochondrial dynamics, encompassing continuous fission and fusion, are critical for mesenchymal stem cell (MSC) differentiation.^9–11^ These processes establish a dynamic network that is necessary for the mitochondrial morphology, abundance, and function.^12^^,^^13^ Forni et al. reported an increase in the expression of mitochondrial fusion proteins Mfn1 and Mfn2 during early adipogenic and osteogenic differentiation, which was correlated with observable mitochondrial elongation, whereas the expression of fission proteins Drp1, Fis1, and Fis2 increases during chondrogenesis.^10^ Similarly, BMSCs experience substantial mitochondrial remodeling during both osteogenic and adipogenic differentiation, displaying a fragmented phenotype in late-stage osteogenesis and a fused network in late-stage adipogenesis.^14^ Interestingly, it has been demonstrated that increasing mitochondrial fission speeds up bone production,^15^ while increasing fusion hinders osteogenesis.^16^ Moderate mitochondrial fission helps remove defective mitochondria,^17^ encourages mitochondrial biogenesis,^18^ and distributes healthy mitochondria to locations with high energy requirements.^19^ Drp1 is the core executor of mitochondrial fission, and its activity is precisely regulated by phosphorylation: phosphorylation at Ser616 (eg, by ERK) activates Drp1 and promotes fission,^20^ whereas phosphorylation at Ser637 (eg, by PKA) inhibits its activity.^21^^,^^22^ Nevertheless, a crucial unresolved topic is how upstream signaling pathways precisely regulate Drp1 phosphorylation to initiate beneficial mitochondrial fission during BMSC osteogenic development.

The brain-type creatine kinase B (Ckb), an isoform of creatine kinase, maintains cellular energy homeostasis by catalyzing reversible ATP-creatine phosphorylation.^23^ It is involved in adipocyte thermogenesis,^24^ tumor progression,^25^ and T cell immunity.^26^ Inhibition of Ckb causes cellular senescence in human bronchial epithelial cells (HBECs), positioning Ckb as an important regulator of cellular senescence.^27^ CKB is specifically expressed in jaw mesenchymal cells, according to our analysis of a public single-cell RNA sequencing dataset.^28^ This finding is supported by immunofluorescence (IF) co-localization with the MSC marker Sca-1 in mouse jaw tissues, indicating that Ckb may regulate MSC osteogenic differentiation. Notably, Ckb has been reported to localize to mitochondria,^29^ and has been demonstrated to enhance the production of ATP in the mitochondria by inhibiting the opening of the mitochondrial permeability transition pore (mPTP) and coordinating the reduction of mitochondrial calcium (mCa^2+^) levels.^23^ Given its mitochondrial localization, we hypothesized that Ckb influences osteogenic differentiation by regulating mitochondrial function. Preliminary experiments confirmed that modulating Ckb expression remodels the mitochondrial network and optimizes its function in BMSCs, accompanied by coordinated changes in osteogenic differentiation capacity.

We performed transcriptome sequencing to better understand the mechanism and discovered that overexpression of Ckb led to a substantial upregulation of αB-crystallin (Cryab). Further investigations verified that Ckb and Cryab interacted physically, indicating the creation of a functional complex. Cryab, a small heat shock protein, provides defense against oxidative stress and apoptotic stimuli by acting as a molecular chaperone and signaling regulator.^30^ It can reduce inflammation, apoptosis, and oxidative stress in human bronchial epithelial cells by inhibiting the PI3K-Akt and NF-κB signaling pathways.^31^ Relevant to our research, Cryab is upregulated during the BMSCs osteogenesis, where it interacts with stabilizes β-catenin by preventing its ubiquitination and degradation. This enhances osteogenic differentiation and promotes Wnt signaling.^32^ Moreover, Cryab deficiency in mice leads to increased apoptosis and mitochondrial dysfunction.^33^ These findings collectively suggest that Ckb-Cryab complex may regulate osteogenic differentiation by modulating mitochondrial function.

In summary, this research aims to clarify a unique signaling pathway wherein the Ckb-Cryab complex promotes osteogenic differentiation by regulating mitochondrial dynamics. This finding not only deepens our understanding of the energetic basis of bone metabolism but also provides new insights into the molecular regulation of bone regeneration.

## Methods

The information for all reagents is listed in Table S1.

### Cell isolation, culture, and identification

All experimental procedures were approved by the Ethics Committee of Nanjing Medical University (IACUC-2112018). Four-week-old male C57BL/6J mice were randomly selected for this study. Following euthanasia via anesthetic overdose, the femurs and tibias were dissected. The bone marrow cavity was irrigated to acquire a complete bone marrow suspension. The suspension was centrifuged to eliminate the supernatant. The cells were resuspended in complete medium, consisting of α-MEM (Gibco, USA) supplemented with 10% fetal bovine serum (FBS, NEWZERUM, New Zealand) and 1% penicillin/streptomycin (NCM Biotech, China). The culture medium was replaced every 3 days. Following incubation with antibodies targeting specific surface markers (CD29, CD44, CD45, and CD11b), cells were evaluated via flow cytometry.

### Osteogenic differentiation of mBMSCs

At 70%-80% confluence, the medium was switched to osteogenic induction medium (adding 10 mM β-glycerophosphate (Gibco, USA), 300 μM ascorbic acid (Gibco, USA), and 0.1 μM dexamethasone (Sigma-Aldrich, USA) to the complete medium). The osteogenic induction medium was replaced at intervals of 2-3 days.

### Lentiviral transduction

Lentiviruses for Ckb knockdown (Ckbsh) and Ckb overexpression (Ckb OE), as well as their respective controls (scrambled shRNA [consh] and empty vector), were provided by GeneChem Co. (Shanghai, China). Lentiviral transduction was conducted when cells attained 40%-50% confluence. Multiplicities of infection (MOI) of 0, 10, 20, 40, 60, and 80 were evaluated, and the corresponding virus volume was determined based on the viral titer. The appropriate amount of virus was added to α-MEM medium containing 5 μg/mL polybrene as a transduction enhancer. The medium was refreshed after 12 h. Selection was subsequently conducted utilizing puromycin (2.5 μg/mL) for a duration of 5 days. The optimal MOI was determined, and transduction efficiency was verified.

### Alkaline phosphatase staining and alizarin red S staining

After 48 h of lentiviral transduction, the complete medium was replaced with osteogenic induction medium. Following 7 days period of osteogenic induction, alkaline phosphatase (ALP) staining was conducted utilizing a BCIP/NBT ALP Color Development Kit (Beyotime, China).

After 14 days of osteogenic induction, calcium nodule deposition was observed under an optical microscope. The cells were incubated in Alizarin Red S (AR-S) staining solution (Leagene Biotechnology, China) for 15 minutes. Subsequent to washing, the plates were scanned. Following scanning, a 10% solution of cetylpyridinium chloride (CPC) solution was added. After the calcium nodules were completely dissolved, the optical density (OD) was measured.

### Protein extraction

RIPA lysis buffer (Beyotime, China) was added to lyse the cells. The cell lysate was collected and subjected to ultrasonic disruption under ice-bath conditions. Subsequent to ultrasonication, the lysate was centrifuged. Following centrifugation, the supernatant was collected. The residual protein samples were combined with loading buffer (NCM Biotech, China), followed by boiling at 100 °C for 10 minutes. The denatured protein samples can be stored at −80 °C for subsequent experiments.

### Western blotting

SDS-PAGE gels (Yeasen, China) of appropriate concentrations were selected for protein electrophoresis. Following electrophoresis, semi-dry transfer was conducted onto a poly-vinylidene difluoride (PVDF) membrane (Millipore, USA). Subsequent to transfer, the membrane was blocked with 5% skim milk and then sequentially incubated with primary antibody (4 °C, overnight) and HRP-conjugated secondary antibody (room temperature, 1 hour). Protein bands were visualized using ECL luminescent reagent (Abbkine, China) and detected via a chemiluminescence imaging system (Thermo Fisher Scientific, USA).

### Co-immunoprecipitation

According to the manufacturer’s instructions, the extracted proteins were incubated with the primary antibody and subsequently mixed with the Protein A/G magnetic beads (TOPSCIENCE, China). Finally, loading buffer was incorporated, and the mixture was heated at 95 °C for a duration of 5 minutes. After magnetic separation, the supernatant was collected.

### Quantitative real-time PCR

Cells were collected for total RNA extraction utilizing a commercial kit (Vazyme, China). The extracted RNA was subsequently reverse-transcribed into complementary DNA (cDNA) with a Primescript RT reagent kit (Vazyme, China). Quantitative real-time PCR (qRT-PCR) analyses were conducted on a Quant Studio 7 system (Applied Biosystems, USA) with Taq Pro Universal SYBR qPCR Master Mix (Vazyme, China). Expression levels were standardized to β-actin. The primer sequences are detailed in Table S2.

### JC-1 assay

JC-1 (Beyotime, China), a fluorescent probe extensively utilized for assessing mitochondrial membrane potential (ΔΨm), is appropriate for evaluations in cells, tissue samples, and isolated mitochondria. Following a 20-minute incubation with the JC-1 working solution at 37 °C in the dark, the cells underwent 2 washes with the specific staining buffer. Observations were conducted using a fluorescence microscope. Green fluorescence denotes the monomeric form of JC-1, while red fluorescence signifies the aggregated form. For quantitative analysis of mitochondrial membrane potential, the ratio of red (aggregate) to green (monomeric) fluorescence intensity was calculated using ImageJ software. At least 3 randomly selected fields per sample were analyzed, and the average ratio was calculated for each group.

### Mitochondrial superoxide assay (MitoSOX)

After being cultured for the predetermined number of days, the initial culture medium in the wells was removed according to the manufacturer’s instructions of the MitoSOX Red Mitochondrial Superoxide Assay Kit (Beyotime, China). The samples were then incubated with the staining working solution by incubation at 37 °C in the dark for 20 minutes. The nuclei were dyed using Hoechst dye (Beyotime, China), and the samples were promptly examined using a fluorescence microscope.

### ATP assay

Intracellular ATP concentrations were quantified utilizing an Enhanced ATP Assay Kit (Beyotime, China) according to the manufacturer’s instructions. BMSCs were lysed using the supplied lysis buffer, and the lysates were centrifuged at 12 000 × g for 5 minutes at 4 °C. The supernatant was collected and mixed with ATP detection working solution, after which luminescence was promptly quantified using a luminometer. ATP levels were standardized to total protein concentration and expressed as nmol/mg protein.

### Oxygen consumption rate assay

Oxygen consumption rate (OCR) was assessed utilizing an Oxygen Consumption Rate Fluorescence Assay Kit (Elabscience, China) following the manufacturer’s instructions. BMSCs were seeded in 96-well black clear-bottom plates. Subsequent to treatment, cells were rinsed with pre-warmed assay buffer, and the oxygen-sensitive probe was added. Fluorescence was quantified every 2 minutes for 90 minutes using a fluorescence microplate reader (excitation/emission = 405/650 nm). The OCR was calculated as the slope of fluorescence intensity over time (ΔF/min) during the linear phase, following the subtraction of background from cell-free controls.

### Mdivi-1 treatment

Mdivi-1 (MedChemExpress, USA) was dissolved in dimethyl sulfoxide (DMSO) as a stock solution and stored at −20 °C. To ascertain the appropriate working concentration, cell counting kit-8 (CCK-8) assays were conducted utilizing various concentrations of Mdivi-1 (0, 2.5, 5, 10, 25, 50, and 100 μM). Based on cell viability (>90%, *P* > .05 vs. control), 5 μM was selected for subsequent experiments. Mdivi-1 was added to the culture medium at the initiation of osteogenic induction and renewed every 2 days.

### siRNA transfection

Small interfering RNA (siRNA) targeting Drp1 and negative control siRNA (si-NC) were purchased from GeneChem Co. (Shanghai, China). BMSCs were seeded in 6-well plates and cultured until achieving 60%-70% confluence. Transfection was conducted using Lipofectamine 3000 (Invitrogen, USA) according to the manufacturer’s protocol. siRNA and Lipofectamine 3000 were individually diluted in α-MEM, mixed, and incubated for 15 min at room temperature. The mixture was then added to the cells to a final siRNA concentration of 50 nM. After 48 hour, cells were collected to validate Drp1 knockdown using qRT-PCR and Western blot, and were subsequently utilized for osteogenic induction experiments.

### Animal experiment design

#### Ethical approval and experimental animals

This study was approved by the Institutional Animal Care and Use Committee (IACUC) of Nanjing Medical University (IACUC-2112018). Forty-eight 4-week-old male C57BL/6J mice were selected for the experiment, all of which were purchased from the Animal Experiment Center of Nanjing Medical University.

#### Experimental groups and procedures

The mice were randomly allocated into 4 experimental groups and received tail vein injections of either consh, ckbsh, vector, or ckb OE lentivirus (100 μL, 1 × 10^8^ TU/mL) twice with 1-week interval between injections. Two weeks subsequent to the final injection, the bilateral maxillary first molars were extracted. The mice were euthanized at 7, 14 days post-extraction.

#### Sample collection and processing

Maxillary bone tissues, femurs, and tibias were harvested. Maxillary bone tissues were fixed in 4% paraformaldehyde for 24 hours. BMSCs were isolated from the femurs and tibias to verify the lentiviral infection efficiency.

### Micro-computed tomography

The samples were fixed in specialized CT sample tubes and scanned using a Micro-computed tomography (Micro-CT) system (Micro-CT50, Scanco, Switzerland). The scanning parameters were set as follows: X-ray tube current 72 μA, voltage 55 kV, spatial resolution 15.6 μm, scanning angle 360°, and exposure time 840 seconds. Following scanning, the acquired 2-dimensional image sequences were reconstructed and analyzed in 3 dimensions using DataViewer software. Quantitative analysis of static bone morphometric parameters was performed using CTAn software (version 1.15.4.0, Bruker MicroCT NV, Germany).

### Hematoxylin and eosin (H&E) and Masson staining

The samples were immersed in a 10% ethylenediaminetetraacetic acid (EDTA, pH 7.4) decalcification solution for 1 month. Decalcified samples were dehydrated, paraffin-embedded, and sectioned. Following this, the resulting sections were deparaffinized in xylene and processed through a graded ethanol series (100%, 95%, 85%, and 75%) for rehydration. Subsequently, the sections were stained with hematoxylin and eosin (H&E) and Masson’s Trichrome stain. Bone healing in the mouse tooth extraction sockets was observed and evaluated under a light microscope.

### Immunofluorescence

#### IF for tissue sections

Following deparaffinization and rehydration, antigen retrieval for the paraffin sections was performed by heating in citrate buffer (95 °C) for 10 minutes, with subsequent slow cooling. The sections were blocked with goat serum, then sequentially incubated with primary antibody (4 °C, overnight) and secondary antibody (room temperature, 1 hour), with thorough washing between each step. The sections were mounted with an anti-fade mounting medium containing DAPI (Beyotime, China) and images were acquired under a fluorescence microscope.

#### IF for cells

BMSCs on coverslips were fixed, permeabilized, and blocked with 5% goat serum. Cells were incubated with primary antibodies (4 °C, overnight), followed by fluorescent anti-mouse/rabbit IgG secondary antibodies (room temperature, 1 hour). Nuclei were stained with DAPI. Images were acquired using a fluorescence or confocal microscope.

For co-localization analysis, cells were co-stained with combinations of primary antibodies as indicated. Fluorescence intensity profile analysis was performed using ImageJ software. A straight line was drawn across the region of interest, and the pixel intensities of each channel along the line were plotted. The resulting intensity profiles were used to evaluate the spatial overlap between different signals.

### Bulk RNA sequencing

Transcriptome sequencing and subsequent data analysis were performed by Bio Co. (Shanghai, China) to screen and identify alterations in the global RNA expression profile under Ckb overexpression conditions. Following RNA extraction from mBMSCs in the vector and Ckb OE groups, library-quality RNA was assessed for integrity (Agilent 2100 Bioanalyzer/4200 TapeStation), quantified (Qubit 2.0 Fluorometer), and checked for purity (NanoDrop ND-2000). Sequencing libraries were subsequently constructed and sequenced on an Illumina NovaSeq 6000 platform in PE150 mode. A comparative transcriptome analysis identified differentially expressed genes (DEGs) using cutoff criteria of |log2FC| > 1 and an adjusted *P-*value < .05.

### Statistical analysis

All statistical analyses were conducted with GraphPad Prism 9 (GraphPad Software, USA), with results shown as the mean ± standard deviation (mean ± SD). Differences between 2 groups were evaluated by unpaired 2-tailed Student’s *t*-test, while comparisons among multiple groups were performed using one-way analysis of variance (ANOVA). In all statistical analyses, a *P-*value of less than 0.05 (*P* < .05) was considered statistically significant.

## Results

### Single-cell transcriptomic analysis of human fetal jaw tissues reveals specific expression of CKB in the jaw mesenchyme

**Figure 1.**
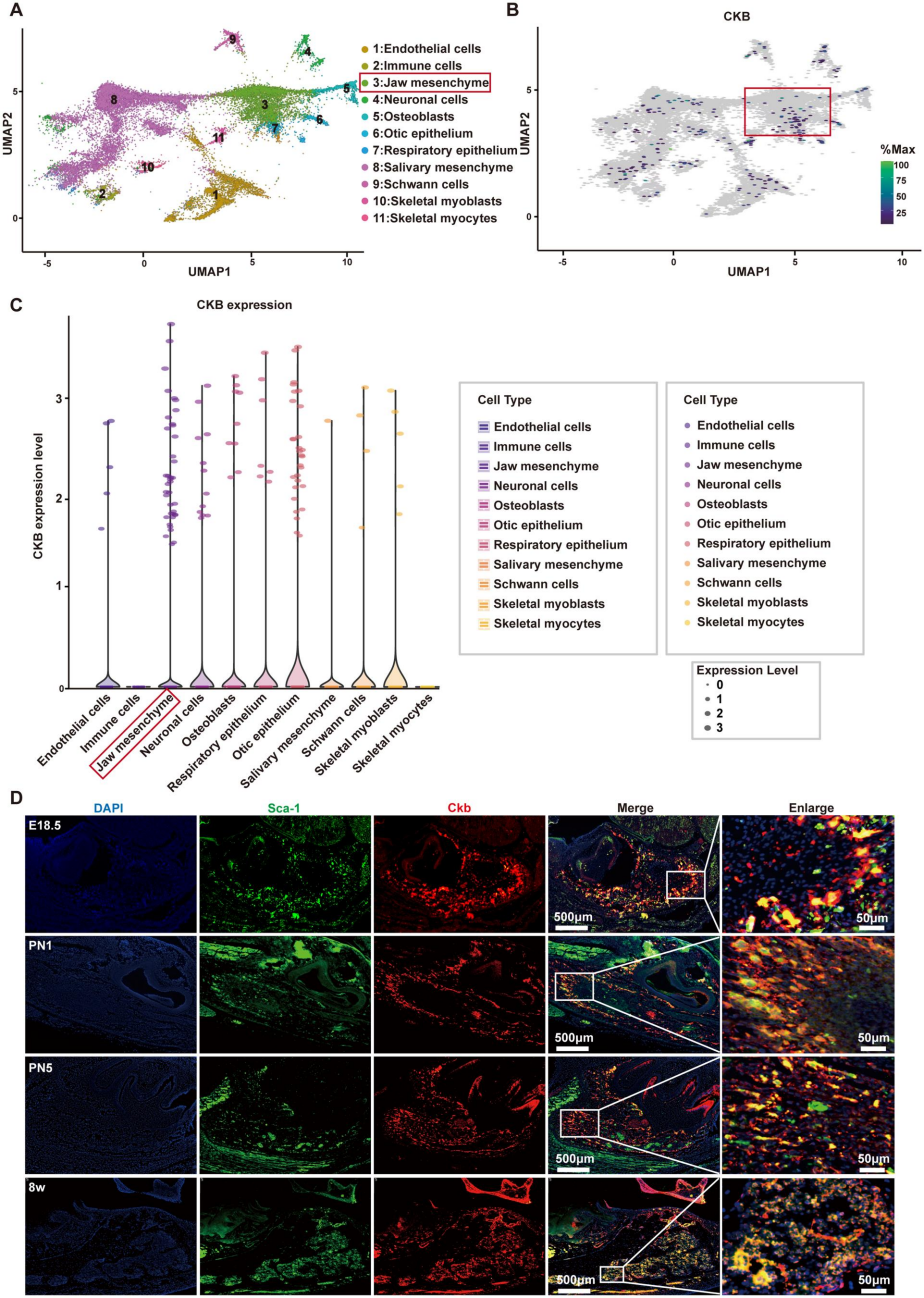
Single-cell transcriptomic analysis of human fetal jaw tissues reveals specific expression of creatine kinase B (CKB) in the jaw mesenchyme. (A) UMAP projection of single-cell RNA sequencing data from human fetal jaw tissues. Cells are categorized by eleven distinct, computationally identified cell populations. (B) UMAP plot showing the expression pattern of CKB across the cell clusters. Note the specific and high expression in the jaw mesenchymal population. (C) Violin plot illustrating the expression level and distribution of CKB transcripts across the annotated cell clusters, demonstrating its notable enrichment in jaw mesenchymal cells. (D) Immunofluorescence co-staining of Ckb (red) and the mesenchymal stem cell marker Sca-1 (green) on sagittal sections of mouse jaw tissues at embryonic day 18.5 (E18.5), postnatal day 1 (PN1), postnatal day 5 (PN5), and adult (8 weeks). Representative images from *n* = 3 mice per group.

To thoroughly delineate the cellular heterogeneity of human fetal jaw tissues, we re-analyzed a publicly available single-cell RNA sequencing dataset derived from developing human tooth and jaw tissues.^28^ The unsupervised clustering of the integrated dataset identified eleven distinct cell populations, including multiple mesenchymal, epithelial, and specialized dental lineages (Figure 1A). The populations were annotated based on established marker genes and their transcriptional profiles, providing a comprehensive cellular census of the developing human jaw.

We subsequently analyzed the expression of *CKB*, a crucial enzyme involved in cellular energy metabolism, within these clusters. Uniform Manifold Approximation and Projection (UMAP) visualization revealed that *CKB* was prominently and specifically expressed in the jaw mesenchyme cluster (Figure 1B). The expression pattern was further confirmed by violin plot analysis, revealing a significant enrichment of *CKB* transcripts in jaw mesenchymal cells compared to other cell types (Figure 1C).

To confirm the spatiotemporal expression of Ckb in vivo and ascertain its cellular localization, we conducted immunofluorescence co-staining of Ckb with the MSC marker Sca-1 on mouse jaw tissues at embryonic day 18.5 (E18.5), postnatal day 1 (PN1), postnatal day 5 (PN5), and adult (8 weeks). In accordance with our transcriptome results, Ckb was highly expressed and co-localized with Sca-1 in the mesenchymal compartment of both the developing and adult jaw (Figure 1D).

Considering that jaw mesenchyme gives rise to BMSCs and that osteogenesis is a highly energy-demanding process, the notable concentration of Ckb in Sca-1+ mesenchymal cells prompted us to propose that Ckb may functionally regulate the osteogenic differentiation of BMSCs.

### Ckb promotes osteogenic differentiation of BMSCs *in vitro*

BMSCs were obtained from 4-week-old male C57BL/6J mice for the research. Flow cytometry analysis demonstrated positive expression of murine MSC surface markers (CD44 and CD29), and negative expression of hematopoietic cell markers (CD45 and CD11b) (Figure S1A). The isolated BMSCs were cultured in osteogenic and adipogenic induction media. Alizarin red S staining (ARS) and Oil Red O staining indicated that the isolated BMSCs have the ability to differentiate into osteoblasts and adipocytes, respectively, confirming the multilineage differentiation capacity characteristic of BMSCs (Figure S1B, C). These results indicate that the cells were indeed BMSCs.

**Figure 2.**
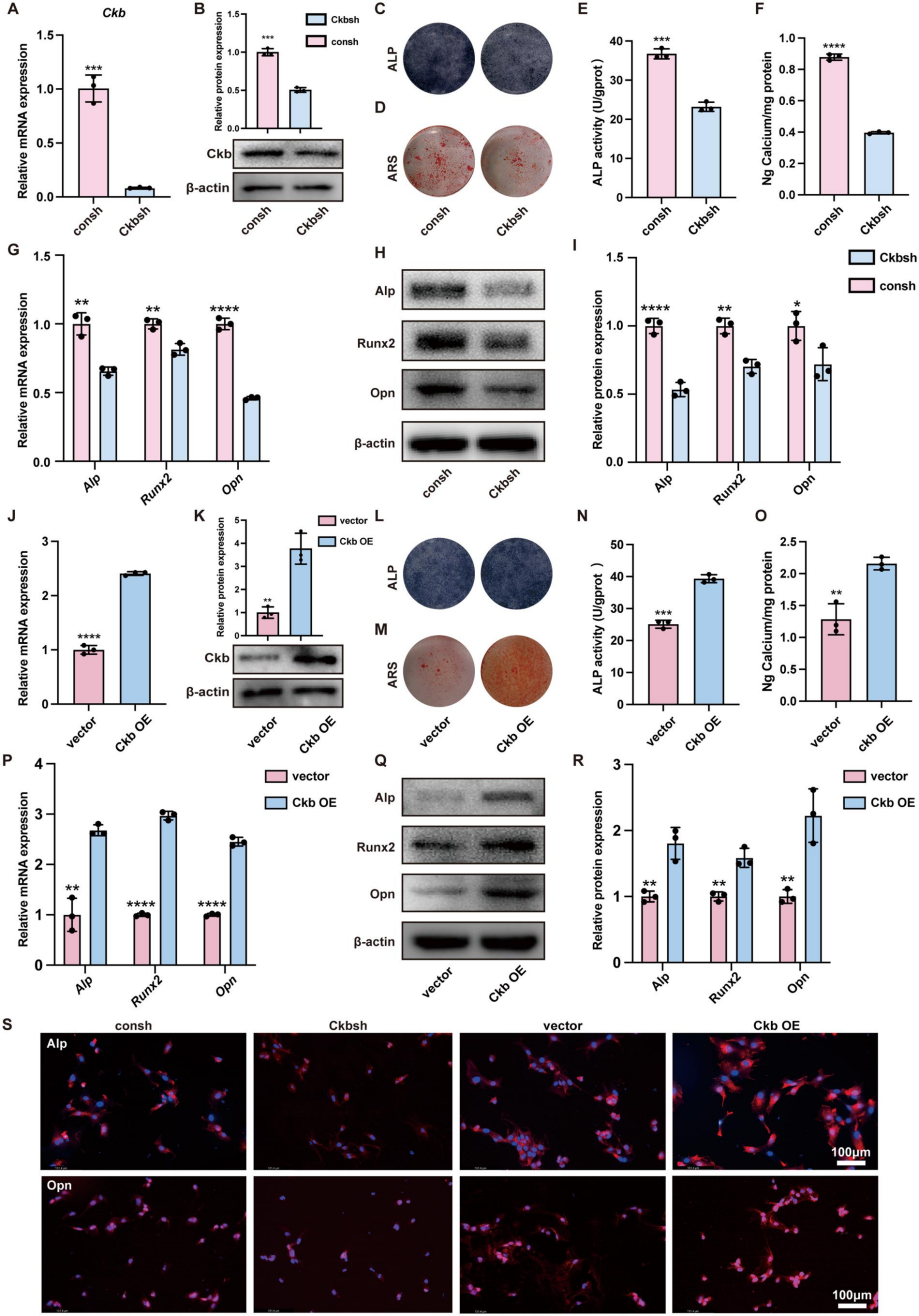
Creatine kinase B (Ckb) promotes osteogenic differentiation of BMSCs in vitro. (A, B) Validation of Ckb knockdown efficiency in bone marrow mesenchymal stem cells (BMSCs) at the mRNA level by quantitative real-time polymerase chain reaction (qRT-PCR) (A) and at the protein level by Western blot (B). (C) Alkaline phosphatase (ALP) staining of BMSCs after 7 days of osteogenic induction. (D) Alizarin Red S staining of BMSCs after 14 days of osteogenic induction. (E) Quantitative analysis of calcium deposition. (F) ALP activity assay. (G-I) Expression of osteogenic markers (Alp, Runx2, and Opn) in Ckb-knockdown BMSCs: mRNA levels via qRT-PCR (G) and protein levels via Western blot (H, I) at 7 days of induction. (J, K) Validation of Ckb overexpression efficiency in BMSCs via qRT-PCR analysis (J) and Western blot (K). (L) ALP staining of BMSCs after 7 days of osteogenic induction. (M) Alizarin Red S staining of BMSCs after 14 days of osteogenic induction. (N) ALP activity assay. (O) Quantitative analysis of calcium deposition. (P-R) Expression of osteogenic markers (Alp, Runx2, and Opn) in Ckb-overexpressing BMSCs: mRNA levels assessed by qRT-PCR (P) and protein levels evaluated by Western blot (Q-R) at 7 days of induction. (S) Immunofluorescence staining of Alp and Opn in BMSCs with Ckb knockdown or overexpression. All data represent mean ± SD. *n* = 3. Statistical significance: *P < .05, **P < .01, ***P < .001, ****P < .0001.

To examine the regulatory function of Ckb in the osteogenic differentiation of BMSCs, we first established stable Ckb-knockdown BMSCs via lentiviral transduction. The knockdown efficiency was validated at both the mRNA and protein levels using qRT-PCR and Western blot, respectively (Figure 2A, B). Functional analysis, including ALP staining, ARS staining, and quantitative assessment of calcium deposition, revealed that Ckb knockdown significantly inhibited osteogenic differentiation (Figure 2C-F). In accordance with this, after 7 days of osteogenic induction, qRT-PCR and Western blot results demonstrated that Ckb knockdown significantly reduced the expression of osteogenic markers, including Alp, Opn, and Runx2, at both the mRNA and protein levels (Figure 2G-I).

Conversely, we generated Ckb-overexpressing BMSCs and confirmed overexpression efficiency (Figure 2J, K). Ckb overexpression facilitated osteogenic differentiation, as evidenced by increased expression of osteogenic markers, elevated ALP activity, and enhanced calcium deposition (Figure 2L-R). Immunofluorescence staining further validated our findings, demonstrating reduced expression of Alp and Opn following Ckb knockdown, and increased expression following Ckb overexpression (Figure 2S). These data collectively emphasize the essential function of Ckb in facilitating the osteogenic differentiation of BMSCs.

### Ckb promotes alveolar bone healing after tooth extraction *in vivo*

**Figure 3.**
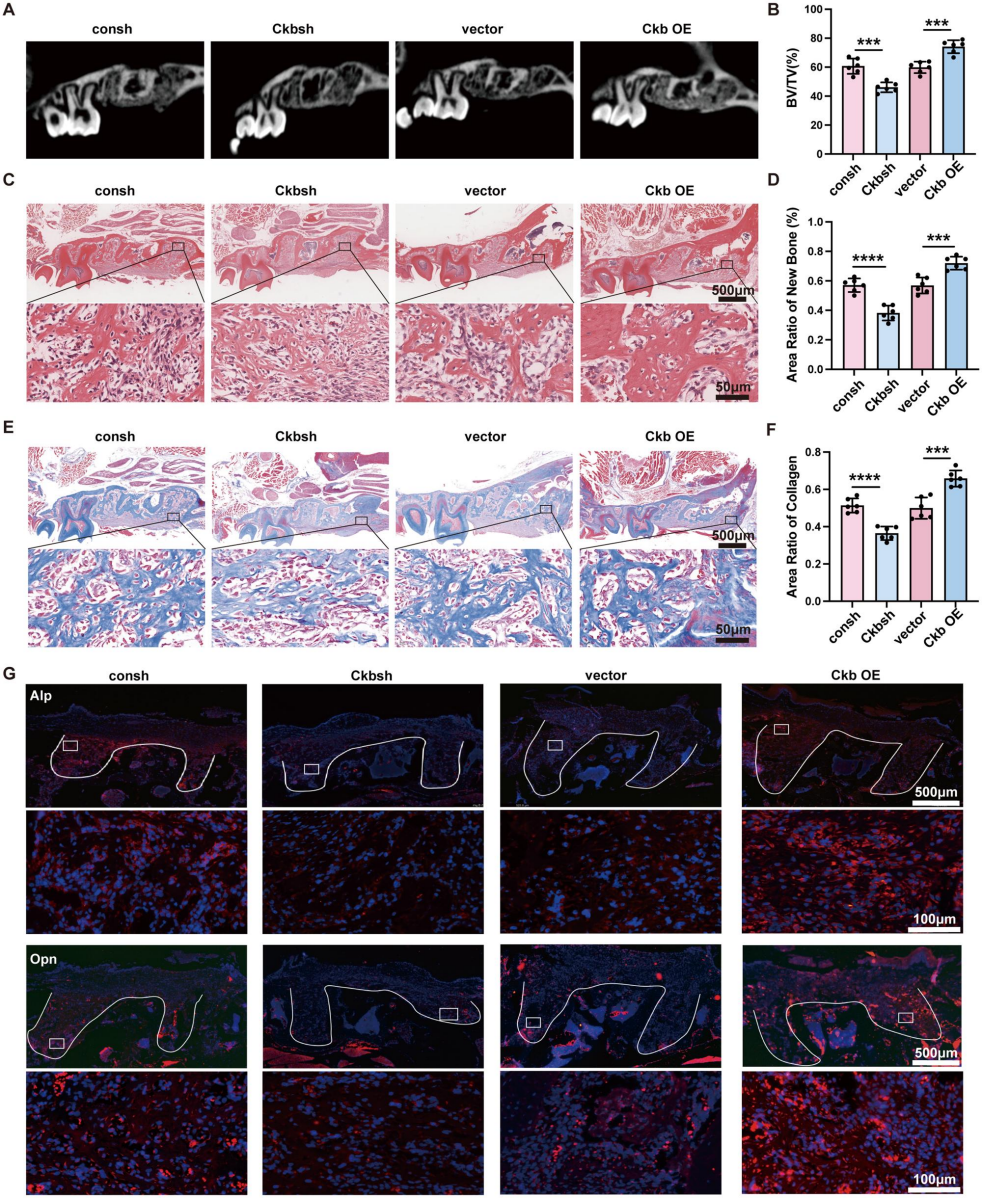
Creatine kinase B (Ckb) promotes alveolar bone healing in a mouse tooth extraction model. (A) Representative micro-CT images of maxillary tooth extraction sockets. (B) Quantitative assessment of the bone volume/tissue volume (BV/TV) ratio in extraction sockets at 7 days post-extraction. (C, D) Hematoxylin and eosin (H&E) staining of extraction sockets at 7 days post-extraction (C) and quantitative analysis of newly formed bone area (D). (E, F) Masson’s trichrome staining of extraction sockets at 7 days post-extraction (E) and quantitative analysis of collagen deposition area (F). (G) Immunofluorescence staining of Alp and Opn in extraction sockets at day 7 post-extraction. All data represent mean ± SD. *n* = 6. Statistical significance: ***P < .001, ****P < .0001.

To further evaluate the in vivo role of Ckb in bone regeneration, we delivered lentiviruses carrying Ckb knockdown (Ckbsh) or Ckb overexpression (Ckb OE) constructs to mice via tail vein injection. mBMSCs were isolated from the femurs and tibias, and effective viral infection was confirmed using Western blot analysis of Ckb protein (Figure S2). Micro-CT analysis of the extraction sockets revealed that the Ckbsh group exhibited significantly impaired bone formation, evidenced by a markedly lower bone volume/total volume (BV/TV) ratio compared to the consh group. In contrast, the Ckb OE group demonstrated enhanced bone healing capacity (Figure 3A, B). Histological assessments, including H&E staining (Figure 3C, D) and Masson’s trichrome staining (Figure 3E, F) further indicated impaired bone regeneration in the Ckbsh group, whereas significant bone regeneration was observed in the Ckb OE group. Immunofluorescence staining results indicated reduced expression of osteogenic markers (Alp, Opn) in the Ckbsh group and increased signal in the Ckb OE group compared to controls (Figure 3G). To further identify the cellular targets of Ckb in vivo, we conducted multiplex immunofluorescence staining on tooth extraction socket tissues. Ckb exhibited significant co-localization with the MSC marker Sca-1, and moderate co-localization with the macrophage marker F4/80 and the endothelial cell marker CD31 (Figure S3A**-**C). Collectively, our in vivo findings indicate that Ckb promotes alveolar bone regeneration following tooth extraction.

### Ckb regulates mitochondrial fusion and fission

**Figure 4.**
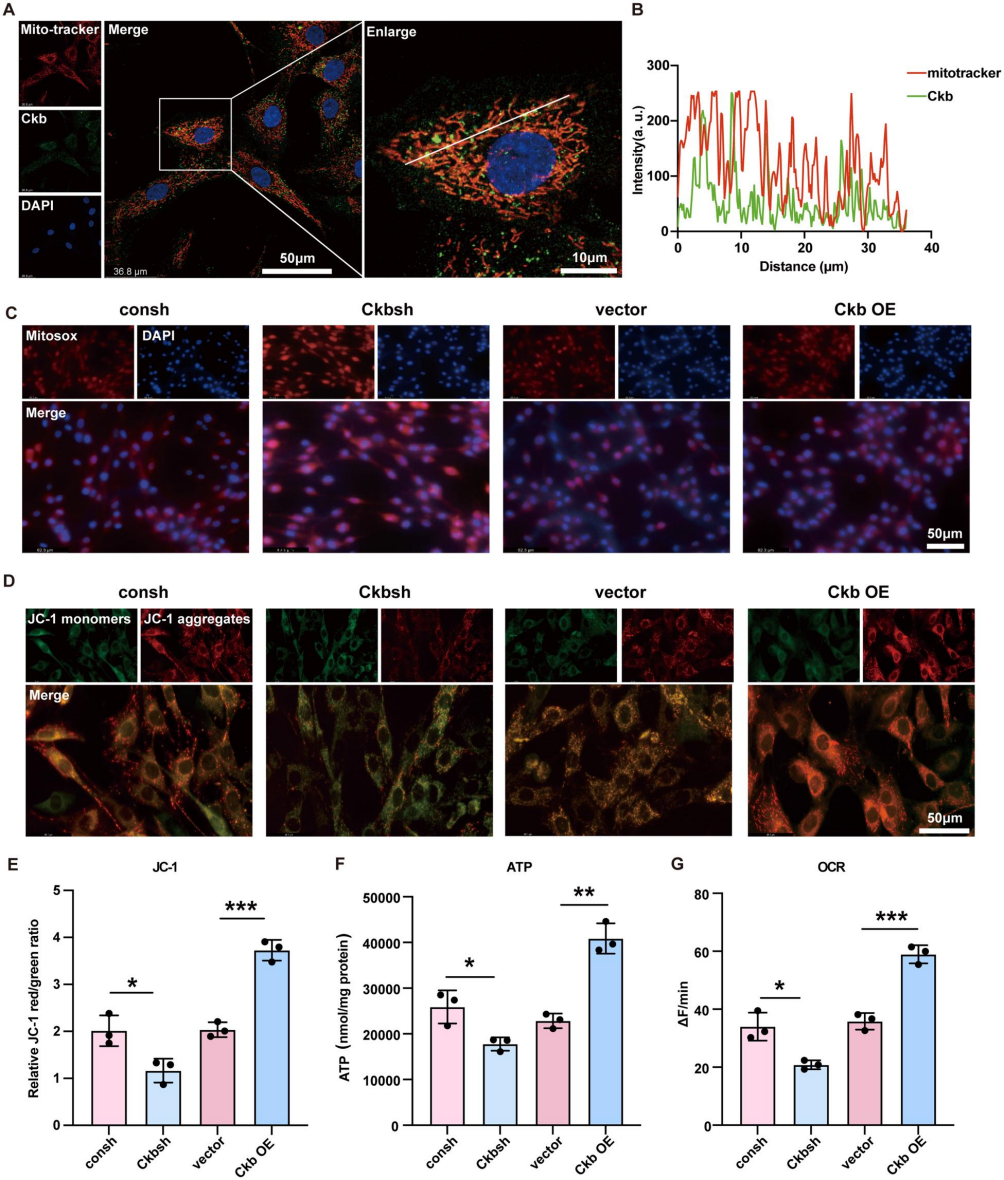
Creatine kinase B (Ckb) regulates mitochondrial function. (A, B) Immunofluorescence co-localization staining of Ckb (green) and mitochondria (red) in bone marrow mesenchymal stem cells (BMSCs) (merge: yellow). (C) MitoSOX Red fluorescence staining of BMSCs showing mitochondrial superoxide levels. (D, E) JC-1 assay to assess mitochondrial membrane potential (ΔΨm): red fluorescence (JC-1 aggregates, and high ΔΨm) and green fluorescence (JC-1 monomers, low ΔΨm) (D) and quantitative analysis of red/green ratio (E). (F) Intracellular adenosine triphosphate (ATP) levels. (G) Oxygen consumption rate (OCR) assay. All data represent mean ± SD. *n* = 3. Statistical significance: *P < .05, **P < .01, ***P < .001.

To investigate the mechanism by which Ckb regulates osteogenesis, we initially assessed its subcellular localization. Co-staining of Ckb and a mitochondrial marker revealed their clear co-localization, indicating the presence of Ckb within mitochondria (Figure 4A, B). Mitochondrial superoxide levels were assessed using MitoSOX staining. The Ckbsh group exhibited higher levels of mitochondrial superoxide compared to the consh group, while the Ckb OE group showed no significant change compared to the vector group (Figure 4C). Assessment of the mitochondrial membrane potential (ΔΨm) showed a positive correlation with Ckb levels: it was elevated in the Ckb OE group and reduced in the Ckbsh group (Figure 4D, E). To further evaluate mitochondrial function, we measured ATP levels and oxygen consumption rate (OCR). Ckb overexpression significantly increased intracellular ATP levels and OCR, while Ckb knockdown markedly reduced both parameters (Figure 4F-G). These results indicate that Ckb enhances mitochondrial respiratory function and ATP production capacity in BMSCs.

**Figure 5.**
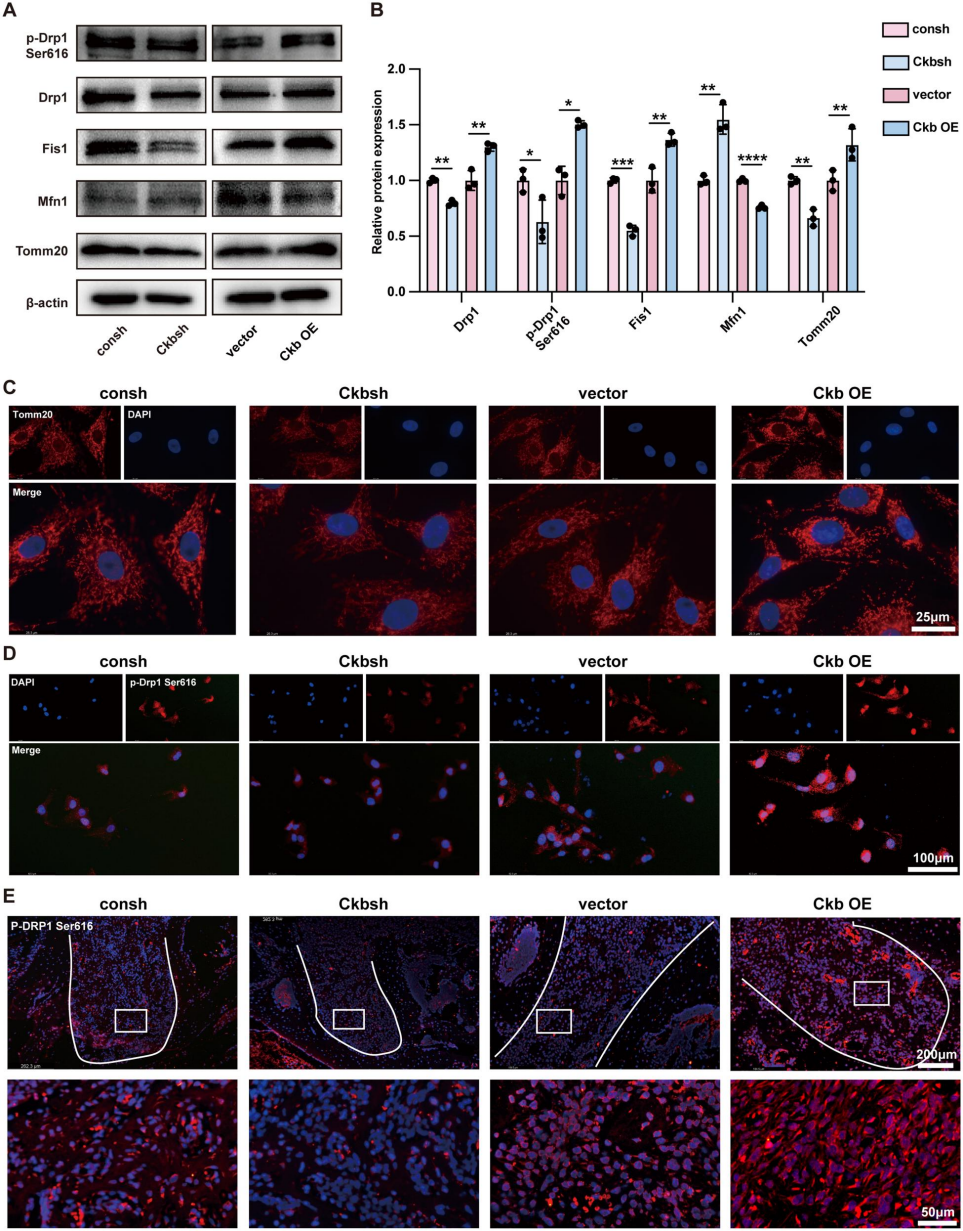
Creatine kinase B (Ckb) regulates mitochondrial fusion and fission in bone marrow mesenchymal stem cells (BMSCs). (A, B) Western blot analysis (A) and quantitative gray value analysis (B) showing the protein expression level of mitochondrial fission/fusion-related proteins (Drp1, P-Drp1 Ser616, Mfn1, Fis1, and Tomm20). (C) Immunofluorescence staining of Tomm20 in BMSCs to visualize mitochondrial morphology. (D) Immunofluorescence staining of p-Drp1 (Ser616) in BMSCs. (E) Immunofluorescence staining of p-Drp1(Ser616) in extraction sockets at day 7 post-extraction. All data represent mean ± SD. *n* = 3. Statistical significance: *P < .05, **P < .01, ***P < .001, ****P < .0001.

Western blot analysis of mitochondrial dynamics proteins indicated that Ckb knockdown downregulated the expression of fission-related proteins (Drp1 and Fis1) and upregulated the fusion-related protein Mfn1. The opposite expression pattern was observed in the Ckb OE group, suggesting that Ckb promotes mitochondrial fission and inhibits fusion (Figure 5A, B). Considering that Drp1 is a core executor of mitochondrial fission and its activity is regulated by phosphorylation (specifically activating phosphorylation at Ser616), we further evaluated Drp1 phosphorylation. Ckb overexpression significantly increased both the total Drp1 protein level and its phosphorylation at the activation site Ser616. In contrast, Ckb knockdown decreased both total Drp1 and p-Drp1(Ser616) levels (Figure 5A, B), indicating that Ckb enhanced both the expression and activity of Drp1.

Morphological analysis of mitochondria via Tomm20 immunofluorescence indicated that mitochondria in the Ckb OE group were shorter, more fragmented, and exhibited increased fluorescence intensity. In contrast, mitochondria in the Ckbsh group displayed an extended, linked tubular network with lower fluorescence intensity (Figure 5C). Furthermore, immunofluorescence staining was conducted on both the cells and the mouse tooth extraction socket bone tissue. The results showed that the staining signal intensity for p-Drp1(Ser616) was decreased in the Ckbsh group, while it was significantly increased in the Ckb OE group (Figure 5D, E). These findings demonstrated that Ckb promotes mitochondrial fission, regulates mitochondrial function, suggesting a potential mechanism through which Ckb regulates osteogenic differentiation.

### Inhibition of mitochondrial fission impairs osteogenic differentiation of BMSCs

**Figure 6.**
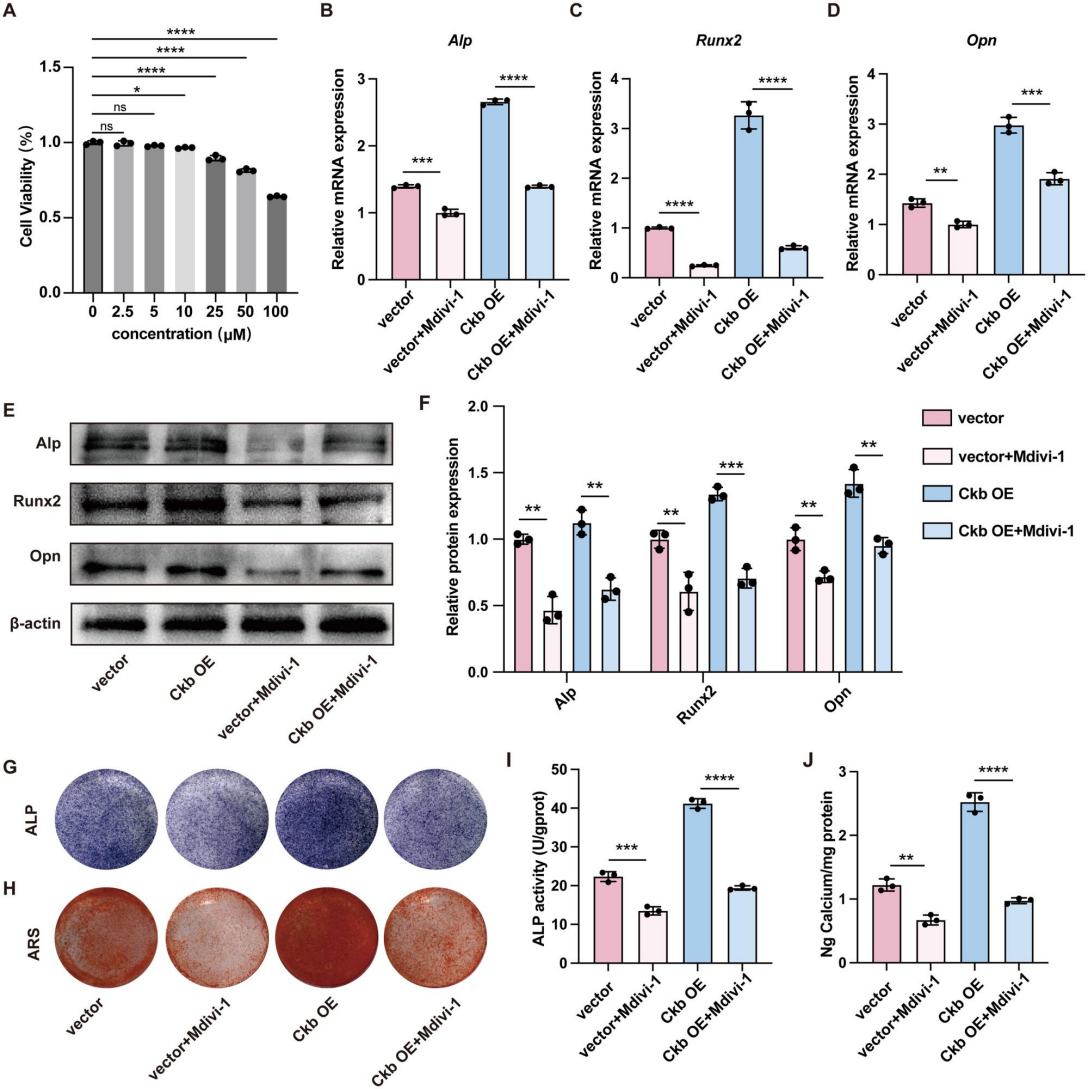
Mdivi-1 treatment impairs creatine kinase B (Ckb)-mediated osteogenic differentiation of bone marrow mesenchymal stem cells (BMSCs). (A) CCK-8 assay evaluated the cytotoxicity of Mdivi-1. (B-D) mRNA expression levels of osteogenesis-related genes (*Alp, Runx2*, and *Opn*) after Mdivi-1 treatment. (E, F) Protein expression levels of osteogenic markers (Alp, Runx2, and Opn) via Western blot (E) and quantitative gray value analysis (F) at 7 days of induction. (G) ALP staining after 7 days of induction. (H) ARS staining after 14 days of induction. (I) Quantitative analysis of ALP activity. (J) Quantitative analysis of calcium deposition. All data represent mean ± SD, *n* = 3. Statistical significance: *P < .05, **P < .01, ***P < .001, ****P < .0001.

To investigate whether mitochondrial fission plays a critical role in Ckb-mediated osteogenic differentiation, we treated the vector and Ckb OE groups with Mdivi-1, a specific inhibitor of mitochondrial fission. CCK-8 assay showed that 5 μM Mdivi-1had no significant effect on cell viability (Figure 6A), and this concentration was used for subsequent experiments. The qRT-PCR results indicated that Mdivi-1 treatment led to a significant downregulation in the mRNA expression levels of osteogenesis-related genes (*Alp, Runx2*, and *Opn*) (Figure 6B-D). The inhibitory effect was additionally validated at the protein level by Western blot analysis (Figure 6E, F). ALP and ARS staining consistently revealed that Mdivi-1 treatment reduced ALP staining intensity and mineralized nodule formation (Figure 6G-J), indicating a significant impairment of osteogenic differentiation potential. These results confirm that mitochondrial fission is a key downstream mechanism through which Ckb promotes osteogenesis.

**Figure 7.**
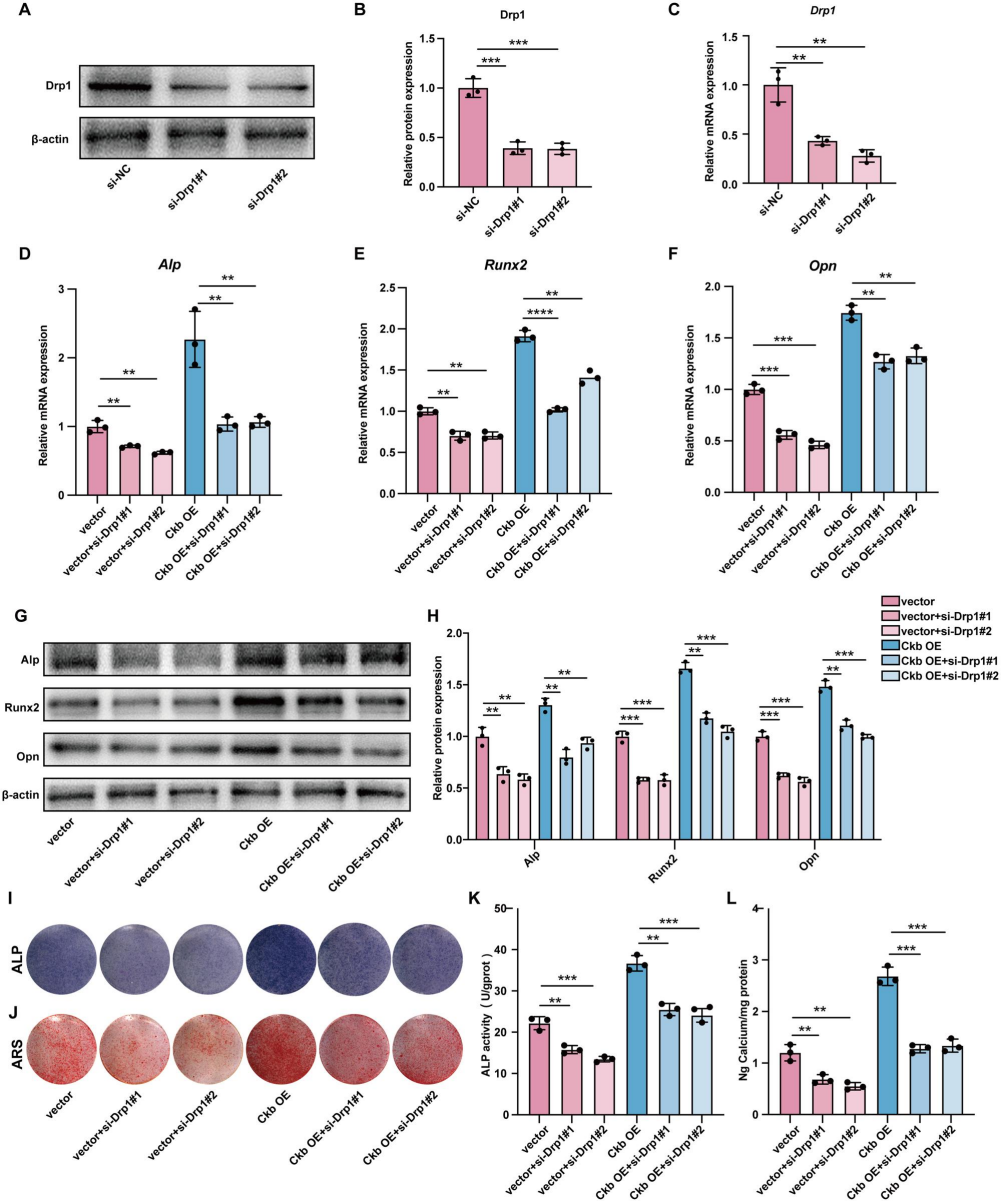
Drp1 knockdown attenuates creatine kinase B (Ckb)-mediated osteogenic differentiation. (A-C) Validation of Drp1 knockdown efficiency by Western blot (A-B) and quantitative real-time polymerase chain reaction (qRT-PCR) (C). (D-F) qRT-PCR analysis of *Alp*, *Runx2*, and *Opn*. (G, H) Western blot analysis (G) and quantitative analysis (H) of Alp, Runx2, and Opn. (I) ALP staining after 7 days of induction. (J) ARS staining after 14 days of induction. (K) Quantitative analysis of ALP activity. (L) Quantitative analysis of calcium deposition. All data represent mean ± SD, *n* = 3. *P < .05, **P < .01, ***P < .001, ****P < .0001.

To further validate the essential role of Drp1 in Ckb-mediated osteogenesis and exclude potential off-target effects of pharmacological inhibition, we employed siRNA to downregulate Drp1 expression in Ckb-overexpressing BMSCs. Western blot and qRT-PCR validated the effective knockdown of Drp1 (Figure 7A-C). Functional experiments demonstrated that Drp1 knockdown significantly reversed the Ckb-induced upregulation of osteogenic markers (Alp, Runx2, Opn) at both mRNA and protein levels (Figure 7D-H), and reduced ALP activity and mineralized nodule formation (Figure 7I-L).

### Cryab regulates osteogenic differentiation and mitochondrial function in BMSCs

**Figure 8.**
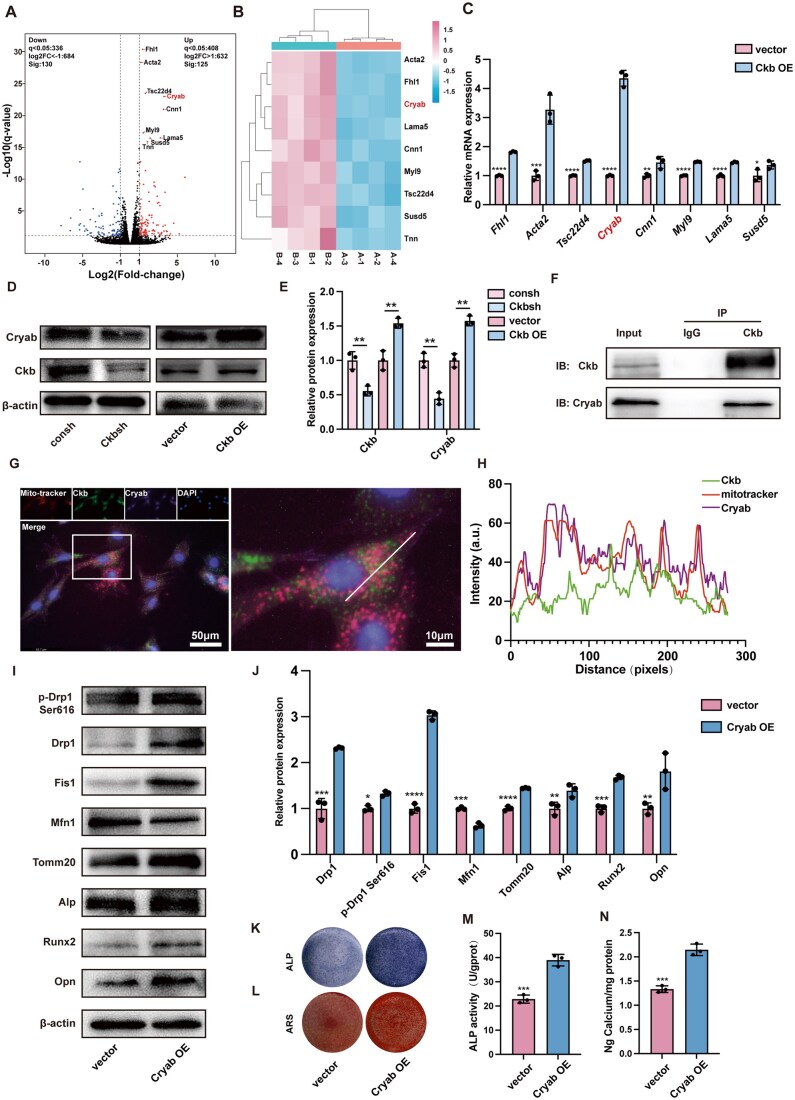
Cryab regulates osteogenic differentiation and mitochondrial function in bone marrow mesenchymal stem cells (BMSCs). (A-B) Transcriptome sequencing results of Ckb-OE BMSCs vs. vector: volcano plot of differentially expressed genes (DEGs; |log2FC| > 1, *P* < .05; A) and heatmap of top DEGs (B). (C) Validation of mRNA expression levels of top 8 DEGs (including *Cryab*) by quantitative real-time polymerase chain reaction (qRT-PCR). (D, E) Western blot analysis (D) and quantitative gray value analysis (E) of Cryab protein levels in BMSCs with Ckb knockdown or overexpression. (F) Co-immunoprecipitation (Co-IP) assay showing interaction between Ckb and Cryab. Ckb was immunoprecipitated, and Cryab was detected by Western blot (input: total protein lysate). (G, H) Immunofluorescence co-localization staining of Ckb, Cryab, and mitochondria in BMSCs. (I, J) Western blot analysis (I) and quantitative analysis (J) of osteogenic markers (Alp, Runx2, and Opn) and mitochondrial dynamics-related proteins (Drp1, p-Drp1 ser616, Fis1, Mfn1, and Tomm20) in Cryab-overexpressing BMSCs. (K) ALP staining of Cryab-overexpressing BMSCs after 7 days of induction. (L) Alizarin Red S staining after 14 days of induction. (M) Quantitative analysis of ALP activity. (N) Quantitative analysis of calcium deposition. All data represent mean ± SD, *n* = 3. Statistical significance: *P < .05, **P < .01, ***P < .001, ****P < .0001.

To further investigate the regulatory networks of Ckb, we conducted transcriptome sequencing on Ckb-overexpressing BMSCs. Differential expression analysis of the sequenced genes, applying thresholds of a *Q*-value ≤ .05 and |log2FC| ≥ 1, identified 255 differentially expressed genes (DEGs), including 125 upregulated and 130 downregulated genes (Figure 8A, B). The top 20 upregulated and downregulated genes are listed in Table S3. Validation of the top 8 DEGs by qRT-PCR revealed that Cryab exhibited the most significant upregulation (Figure 8C). Western blot results corroborated that Cryab expression increased upon Ckb overexpression and decreased upon Ckb knockdown (Figure 8D, E). The co-immunoprecipitation (Co-IP) assay confirmed a protein-protein interaction between Ckb and Cryab (Figure 8F). Furthermore, co-localization staining showed a spatial association among Ckb, Cryab, and mitochondria (Figure 8G, H). These data suggest that Cryab may function within the Ckb pathway to regulate osteogenic differentiation and mitochondrial function.

We then established Cryab*-*overexpressing BMSCs. Western blot analysis showed that Cryab overexpression significantly upregulated the expression of osteogenic markers (Alp, Runx2, and Opn) (Figure 8I, J). It also increased the levels of mitochondrial fission-related proteins (p-Drp1 Ser616, Drp1, and Fis1) and decreased the fusion-related protein Mfn1 (Figure 8I, J). Functionally, Cryab overexpression enhanced ALP activity and promoted matrix mineralization (Figure 8K-N). These results demonstrate that Cryab, like Ckb, promotes both mitochondrial fission and osteogenic differentiation.

### Cryab rescues the impaired osteogenic differentiation and mitochondrial dysfunction induced by Ckb knockdown

**Figure 9.**
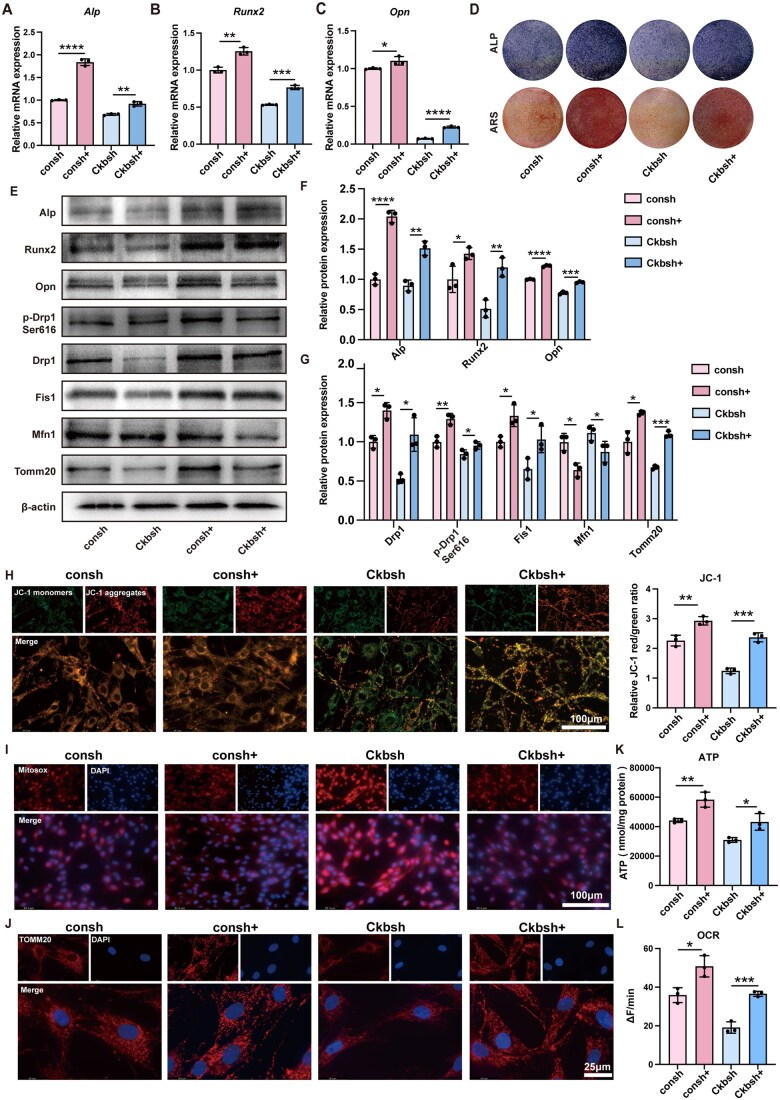
Cryab rescues creatine kinase B (Ckb) knockdown-induced impairmentsin osteogenic differentiation and mitochondrial function of bone marrow mesenchymal stem cells (BMSCs). (A-C) mRNA levels of osteogenic markers (*Alp, Runx2*, and *Opn*) via quantitative real-time polymerase chain reaction (qRT-PCR) at 7 days of osteogenic induction. (D) Alkaline phosphatase (ALP) staining (7 days) and ARS staining (14 days) of BMSCs in each group. (E-G) Western blot analysis (E) and quantitative analysis (F, G) of osteogenic markers (Alp, Runx2, and Opn) and mitochondrial dynamics-related proteins (Drp1, P-Drp1 Ser616, Fis1, and Mfn1). (H) JC-1 staining and quantitative analysis. (I) MitoSOX Red staining showing mitochondrial superoxide levels. (J) Immunofluorescence staining of Tomm20 in BMSCs showing mitochondrial morphology. (K) Intracellular ATP levels. (L) Oxygen consumption rate (OCR). All data are presented as mean ± SD, *n* = 3. Statistical significance: *P < .05, **P < .01, ***P < .001, ****P < .0001.

To ascertain whether Cryab is a critical downstream effector of Ckb, we conducted a rescue experiment by transducing Ckb-knockdown BMSCs with Cryab overexpression lentivirus. qRT-PCR results showed that Cryab overexpression upregulated the mRNA expression levels of osteogenesis-related genes *(Alp, Runx2*, and *Opn)* (Figure 9A-C). ALP and ARS staining further demonstrated that Cryab overexpression rescued the impaired osteogenic differentiation (Figure 9D). Western blot analysis revealed a consistent recovery at the protein level for both osteogenic markers and mitochondrial dynamics proteins, with Cryab restoring the promotion of mitochondrial fission (Figure 9E-G). Functionally, Cryab overexpression rescued the decrease in mitochondrial membrane potential (Figure 9H) and the increased mitochondrial superoxide levels caused by Ckb knockdown (Figure 9I). Morphologically, Tomm20 staining revealed that Cryab overexpression reversed the elongated mitochondrial network phenotype in Ckb-knockdown cells, resulting in more fragmented and numerous mitochondria with higher fluorescence intensity (Figure 9J). Furthermore, we assessed ATP levels and OCR to determine whether Cryab could rescue the energy metabolism defects caused by Ckb knockdown. Ckb knockdown significantly reduced intracellular ATP levels and OCR, and Cryab overexpression effectively reversed these deficits (Figure 9K, L). These results demonstrate that Cryab can rescue both the osteogenic differentiation dysfunction and the mitochondrial dysfunction induced by Ckb knockdown.

## Discussion

This study identified a novel mechanism whereby the Ckb-Cryab complex promotes osteogenic differentiation of BMSCs and bone regeneration through the regulation of mitochondrial dynamics. We demonstrated that this complex enhances Drp1 phosphorylation at Ser616, thereby optimizing mitochondrial fission. This fission, in turn, optimizes mitochondrial function, likely by facilitating the removal of damaged components.^34^ Our findings align with recent reports that osteogenic induction triggers mitochondrial fragmentation to accelerate bone regeneration.^15^ The observed shift in dynamics, characterized by increased fission and a trend toward suppressed fusion (Mfn1), suggests that the Ckb-Cryab complex not only activates Drp1 but may also coordinate a broader mitochondrial remodeling program to meet the energy and metabolic requirements of osteogenesis.

A significant discovery is the physical interaction between Ckb and Cryab, revealed through Co-IP and rescue experiments. We further established that this complex regulates Drp1 phosphorylation, thereby influencing both the osteogenic differentiation capacity and mitochondrial function of BMSCs. As a small heat shock protein, Cryab primarily functions as a molecular chaperone, binding diverse “client proteins” via its hydrophobic surfaces to prevent their misfolding and modulate activity.^30^ Research indicated that Cryab interacts with signaling proteins, including caspase-3, Bax, and Bcl-xS, thereby suppressing stress-induced apoptosis by preventing their mitochondrial translocation.^35^^,^^36^ Cryab also participates in several kinase signaling pathways.^30^^,^^37^ However, the identification of Ckb as a client protein of Cryab is a novel finding.

Recent study suggests that, in addition to its role in energy buffering, Ckb can function as a protein kinase in some contexts.^38^^,^^39^ However, our study does not provide evidence that the Ckb-Cryab complex directly phosphorylates Drp1. To explore potential indirect mechanisms, we analyzed the phosphorylation levels of Erk, Cdk1, and Drp1 at Ser637. No significant changes in Erk, p-Erk, Cdk1, p-Cdk1, or p-Drp1 Ser637 were observed upon Ckb or Cryab modulation (Figure S4), indicating that the Ckb-Cryab complex does not function via these classical pathways. The precise molecular mechanism by which this complex enhances Drp1 Ser616 phosphorylation remains to be elucidated. Future studies, including in vitro kinase assays with purified proteins, are needed to elucidate whether and how the Ckb-Cryab complex regulates Drp1 phosphorylation.

Although our in vivo studies illustrated the pro-osteogenic function of Ckb in bone regeneration, some limitations should be recognized. First, the in vivo experiments primarily served as functional validation rather than direct mechanistic exploration. Second, systemic lentiviral delivery may affect multiple cell types. Consistent with this, immunofluorescence staining revealed that Ckb is expressed not only in BMSCs (Sca-1+) but also in macrophages (F4/80+) and endothelial cells (CD31+) within the healing tissue (Figure S3). Given that macrophages and endothelial cells engage with BMSCs to coordinate bone regeneration (eg, macrophages secrete cytokines that regulate BMSC osteogenic differentiation,^40^^,^^41^ and endothelial cells support bone formation through angiogenesis^42^), the potential contribution of Ckb in these cell types to the observed bone healing phenotype cannot be excluded. Therefore, the observed bone healing phenotype cannot be solely attributed solely to BMSCs. Future studies employing cell-specific conditional knockout mice and co-culture systems are needed to elucidate the cell-autonomous functions of Ckb and its potential role in intercellular communication during bone regeneration.

It is also worth noting that the initial single-cell data originated from human fetal jaw tissues, while our functional validation was conducted in adult mouse BMSCs. Fetal jaw mesenchyme and adult bone marrow-derived BMSCs differ in their developmental stages and anatomical origins. However, these cell types are developmentally related, as both derive from the mesenchymal lineage. Furthermore, it has been documented that embryonic skeletal development and adult bone repair exhibit conserved molecular mechanisms, with several genes involved in embryogenesis similarly expressed during adult bone regeneration.^43^^,^^44^ Thus, the notable enrichment of Ckb in fetal jaw mesenchyme prompted us to explore its potential role in adult BMSCs. Our subsequent observation that Ckb is expressed in adult mouse BMSCs and promotes their osteogenic differentiation is consistent with this notion.

## Conclusion

In summary, our research demonstrated that Ckb interacts with Cryab to form a functional complex that enhances the osteogenic differentiation of BMSCs by regulating mitochondrial dynamics. Furthermore, in vivo investigations indicate that Ckb promotes bone regeneration in mice. Collectively, these findings provide new insights into the molecular regulation of bone regeneration.

## Supplementary Material

szag056_Supplementary_Data

## Data Availability

The datasets generated and analyzed during this study are available from the corresponding author upon reasonable request.

## References

[1] Xu H , Zhang G, Xu K, et al Mussel-inspired dual-functional PEG hydrogel inducing mineralization and inhibiting infection in maxillary bone reconstruction. Mater Sci Eng C Mater Biol Appl. 2018;90:379-386. 10.1016/j.msec.2018.04.066 29853103

[2] Ni Y , Lu P, Yang Z, et al The application of fibular free flap with flexor hallucis longus in maxilla or mandible extensive defect: a comparison study with conventional flap. World J Surg Oncol. 2018;16:149. 10.1186/s12957-018-1450-2 30037329 PMC6057000

[3] Zhao J , Zhou YH, Zhao YQ, et al Oral cavity-derived stem cells and preclinical models of jaw-bone defects for bone tissue engineering. Stem Cell Res Ther. 2023;14:39. 10.1186/s13287-023-03265-z 36927449 PMC10022059

[4] Raposo-Amaral CE , Bueno DF, Almeida AB, et al Is bone transplantation the gold standard for repair of alveolar bone defects? J Tissue Eng. 2014;5:2041731413519352. 10.1177/2041731413519352 24551445 PMC3924878

[5] Li B , Shi Y, Liu M, et al Attenuates of NAD(+) impair BMSC osteogenesis and fracture repair through OXPHOS. Stem Cell Res Ther. 2022;13:77. 10.1186/s13287-022-02748-9 35193674 PMC8864833

[6] Pittenger MF , Mackay AM, Beck SC, et al Multilineage potential of adult human mesenchymal stem cells. Science (1979). 1999;284:143-147. 10.1126/science.284.5411.143 10102814

[7] Uccelli A , Moretta L, Pistoia V. Mesenchymal stem cells in health and disease. Nat Rev Immunol. 2008;8:726-736. 10.1038/nri2395 19172693

[8] Zhao SJ , Kong FQ, Jie J, et al Macrophage MSR1 promotes BMSC osteogenic differentiation and M2-like polarization by activating PI3K/AKT/GSK3β/β-catenin pathway. Theranostics. 2020;10:17-35. 10.7150/thno.36930 31903103 PMC6929615

[9] Li Q , Gao Z, Chen Y, Guan MX. The role of mitochondria in osteogenic, adipogenic and chondrogenic differentiation of mesenchymal stem cells. Protein Cell. 2017;8:439-445. J 10.1007/s13238-017-0385-7 28271444 PMC5445026

[10] Forni MF , Peloggia J, Trudeau K, Shirihai O, Kowaltowski AJ. Murine mesenchymal stem cell commitment to differentiation is regulated by mitochondrial dynamics. Stem Cells (1981). 2016;34:743-755. 10.1002/stem.2248 PMC4803524 26638184

[11] Ren L , Chen X, Chen X, Li J, Cheng B, Xia J. Mitochondrial dynamics: fission and fusion in fate determination of mesenchymal stem cells. Front Cell Dev Biol. 2020;8:580070. 10.3389/fcell.2020.580070 33178694 PMC7593605

[12] Bereiter-Hahn J , Vöth M. Dynamics of mitochondria in living cells: shape changes, dislocations, fusion, and fission of mitochondria. Microsc Res Tech. 1994;27:198-219. 10.1002/jemt.1070270303 8204911

[13] Merz S , Hammermeister M, Altmann K, Dürr M, Westermann B. Molecular machinery of mitochondrial dynamics in yeast. Biol Chem. 2007;388:917-926. 10.1515/bc.2007.110 17696775

[14] Liu J , Bao X, Huang J, et al TMEM135 maintains the equilibrium of osteogenesis and adipogenesis by regulating mitochondrial dynamics. Metabolism. 2024;152:155767. 10.1016/j.metabol.2023.155767 38154611

[15] Suh J , Kim NK, Shim W, et al Mitochondrial fragmentation and donut formation enhance mitochondrial secretion to promote osteogenesis. Cell Metab. 2023;35:345-360.e7. e347 10.1016/j.cmet.2023.01.003 36754021

[16] Deng L , Yi S, Yin X, Li Y, Luan Q. MFN2 knockdown promotes osteogenic differentiation of iPSC-MSCs through aerobic glycolysis mediated by the wnt/β-catenin signaling pathway. Stem Cell Res Ther. 2022;13:162. 10.1186/s13287-022-02836-w 35413941 PMC9006575

[17] Twig G , Elorza A, Molina AJ, et al Fission and selective fusion govern mitochondrial segregation and elimination by autophagy. EMBO J. 2008;27:433-446. 10.1038/sj.emboj.7601963 18200046 PMC2234339

[18] Kleele T , Rey T, Winter J, et al Distinct fission signatures predict mitochondrial degradation or biogenesis. Nature. 2021;593:435-439. 10.1038/s41586-021-03510-6 33953403

[19] Wang C , Du W, Su QP, et al Dynamic tubulation of mitochondria drives mitochondrial network formation. Cell Res. 2015;25:1108-1120. 10.1038/cr.2015.89 26206315 PMC4650629

[20] Kashatus JA , Nascimento A, Myers LJ, et al Erk2 phosphorylation of Drp1 promotes mitochondrial fission and MAPK-driven tumor growth. Mol Cell. 2015;57:537-551. 10.1016/j.molcel.2015.01.002 25658205 PMC4393013

[21] Miao Z , Tian W, Ye Y, et al Hsp90 induces Acsl4-dependent glioma ferroptosis via dephosphorylating Ser637 at Drp1. Cell Death Dis. 2022;13:548. 10.1038/s41419-022-04997-1 35697672 PMC9192632

[22] Zhu X , Deng Z, Cao Y, et al Resveratrol prevents Drp1-mediated mitochondrial fission in the diabetic kidney through the PDE4D/PKA pathway. Phytother Res. 2023;37:5916-5931. 10.1002/ptr.8004 37767771

[23] He L , Lin J, Lu S, et al CKB promotes mitochondrial ATP production by suppressing permeability transition pore. Adv Sci (Weinh). 2024;11:e2403093. 10.1002/advs.202403093 38896801 PMC11336976

[24] Rahbani JF , Bunk J, Lagarde D, et al Parallel control of cold-triggered adipocyte thermogenesis by UCP1 and CKB. Cell Metab. 2024;36:526-540.e7. e527. 10.1016/j.cmet.2024.01.001 38272036

[25] Wang Z , Hulsurkar M, Zhuo L, et al CKB inhibits epithelial-mesenchymal transition and prostate cancer progression by sequestering and inhibiting AKT activation. Neoplasia. 2021;23:1147-1165. 10.1016/j.neo.2021.09.005 34706306 PMC8551525

[26] Samborska B , Roy DG, Rahbani JF, et al Creatine transport and creatine kinase activity is required for CD8(+) T cell immunity. Cell Rep. 2022;38:110446. 10.1016/j.celrep.2022.110446 35235777

[27] Hara H , Araya J, Takasaka N, et al Involvement of creatine kinase B in cigarette smoke-induced bronchial epithelial cell senescence. Am J Respir Cell Mol Biol. 2012;46:306-312. 10.1165/rcmb.2011-0214OC 21980054

[28] Alghadeer A , Hanson-Drury S, Patni AP, et al Single-cell census of human tooth development enables generation of human enamel. Dev Cell. 2023;58:2163-2180.e2169. 10.1016/j.devcel.2023.07.013 37582367 PMC10629594

[29] Rahbani JF , Roesler A, Hussain MF, et al Creatine kinase B controls futile creatine cycling in thermogenic fat. Nature. 2021;590:480-485. 10.1038/s41586-021-03221-y 33597756 PMC8647628

[30] Zhang J , Liu J, Wu J, Li W, Chen Z, Yang L. Progression of the role of CRYAB in signaling pathways and cancers. Onco Targets Ther. 2019;12:4129-4139. 10.2147/ott.S201799 31239701 PMC6553995

[31] Xie S , Wang X. CRYAB reduces cigarette smoke-induced inflammation, apoptosis, and oxidative stress by retarding PI3K/akt and NF-κB signaling pathways in human bronchial epithelial cells. Allergol Immunopathol. 2022;50:23-29. 10.15586/aei.v50i5.645 36086960

[32] Zhu B , Xue F, Li G, Zhang C. CRYAB promotes osteogenic differentiation of human bone marrow stem cells via stabilizing β-catenin and promoting the wnt signalling. Cell Prolif. 2020;53:e12709. J 10.1111/cpr.12709 31638302 PMC6985673

[33] Wang C , Zhang L, Nie Z, et al Mutation of CRYAB encoding a conserved mitochondrial chaperone and antiapoptotic protein causes hereditary optic atrophy. JCI Insight. 2024;10(1):e182209. 10.1172/jci.insight.182209 PMC11721302 39561005

[34] Chan DC. Mitochondrial dynamics and its involvement in disease. Annu Rev Pathol. 2020;15:235-259. 10.1146/annurev-pathmechdis-012419-032711 31585519

[35] Hu WF , Gong L, Cao Z, et al αA- and αB-crystallins interact with caspase-3 and bax to guard mouse lens development. Curr Mol Med. 2012;12:177-187. 10.2174/156652412798889036 22280356

[36] Mao YW , Liu JP, Xiang H, Li DW. Human alphaA- and alphaB-crystallins bind to bax and Bcl-X(S) to sequester their translocation during staurosporine-induced apoptosis. Cell Death Differ. 2004;11:512-526. 10.1038/sj.cdd.4401384 14752512

[37] Liu ES , Raimann A, Chae BT, Martins JS, Baccarini M, Demay MB. c-raf promotes angiogenesis during normal growth plate maturation. Development. 2016;143:348-355. 10.1242/dev.127142 26657770 PMC4725343

[38] Yang B , Zhang W, Sun L, et al Creatine kinase brain-type regulates BCAR1 phosphorylation to facilitate DNA damage repair. iScience. 2023;26:106684. 10.1016/j.isci.2023.106684 37182100 PMC10173731

[39] Wu K , Yan M, Liu T, et al Creatine kinase B suppresses ferroptosis by phosphorylating GPX4 through a moonlighting function. Nat Cell Biol. 2023;25:714-725. 10.1038/s41556-023-01133-9 37156912

[40] Schlundt C , Fischer H, Bucher CH, Rendenbach C, Duda GN, Schmidt-Bleek K. The multifaceted roles of macrophages in bone regeneration: a story of polarization, activation and time. Acta Biomater. 2021;133:46-57. 10.1016/j.actbio.2021.04.052 33974949

[41] Pajarinen J , Lin T, Gibon E, et al Mesenchymal stem cell-macrophage crosstalk and bone healing. Biomaterials. 2019;196:80-89. 10.1016/j.biomaterials.2017.12.025 29329642 PMC6028312

[42] Ramasamy SK , Kusumbe AP, Wang L, Adams RH. Endothelial notch activity promotes angiogenesis and osteogenesis in bone. Nature. 2014;507:376-380. 10.1038/nature13146 24647000 PMC4943529

[43] Ripamonti U. Global morphogenesis regulating tissue architecture and organogenesis. Biomater Adv. 2025;172:214262. J 10.1016/j.bioadv.2025.214262 40054230

[44] Ferguson C , Alpern E, Miclau T, Helms JA. Does adult fracture repair recapitulate embryonic skeletal formation? Mech Dev. 1999;87:57-66. 10.1016/s0925-4773(99)00142-2 10495271

