# Supplementary material for: Ckb-Cryab-Drp1 axis enhances BMSC osteogenic differentiation and mouse alveolar bone healing through mitochondrial fission modulation
Source: Stem Cells Transl Med. 2026 Aug 5;15(8):szag056. doi: 10.1093/stcltm/szag056 (PMC13441178; doi:10.1093/stcltm/szag056)
Supplement: Supplementary file 1 [file szag056_supplementary_data.docx]

**Supplementary Information**

**CKB-CRYAB-DRP1 AXIS ENHANCES OSTEOGENESIS**

**Ckb-Cryab-Drp1 Axis Enhances BMSC Osteogenic Differentiation and Mouse Alveolar Bone Healing Through Mitochondrial Fission Modulation**

Mingxi Wang^1,2,3#^，Long Wang^1,2,3^，Jingchan Wang^1,2,3^，Shan Gao^1,2,3^, Guoqing Li^1,2,3*^, Chunbo Tang^1,2,3*^

1 Department of Dental Implantology, The Affiliated Stomatological Hospital of Nanjing Medical University, Nanjing 210029, China

2 State Key Laboratory Cultivation Base of Research, Prevention and Treatment for Oral Diseases (Nanjing Medical University), Nanjing 210029, China

3 Jiangsu Province Engineering Research Center of Stomatological Translational Medicine (Nanjing Medical University), Nanjing 210029, China

**Correspondence**

Dr. Chunbo Tang, Department of Dental Implantology, The Affiliated Stomatological Hospital of Nanjing Medical University, No. 136, Hanzhong Road, Gulou District, Nanjing, China.

Email: cbtang@njmu.edu.cn

or

Dr. Guoqing Li, Department of Dental Implantology, The Affiliated Stomatological Hospital of Nanjing Medical University, No. 136, Hanzhong Road, Gulou District, Nanjing, China.

Email: [guoqingli@njmu.edu.cn](mailto:guoqingli@njmu.edu.cn)

**Table S1. The sources of reagents**

| **Reagent** | **Company** |
| --- | --- |
| α-MEM medium | Gibco, USA |
| Fetal bovine serum | Gibco, USA |
| Penicillin/Streptomycin solution | NCM Biotech, China |
| L-Ascorbic acid | Gibco, USA |
| β-Glycerophosphate disodium salt | Gibco, USA |
| Dexamethasone | Sigma-Aldrich, USA |
| CD29-PE | BD, USA |
| CD44-FITC | BD, USA |
| CD45-FITC | BD, USA |
| CD11b-PE | BD, USA |
| Alizarin Red S staining solution | Leagene, China |
| Oil red staining kit | Solarbio, China |
| ALP staining kit | Beyotime, China |
| Phosphate-buffered saline | Gibco, USA |
| PMSF | Beyotime, China |
| phosphatase inhibitors | Beyotime, China |
| RIPA lysis buffer | Beyotime, China |
| Cell Complete Lysis Buffer for Western and IP | Beyotime, China |
| Protein A/G magnetic beads | TOPSCIENCE, China |
| Total RNA extraction kit | Vazyme, China |
| Reverse transcription kit | Vazyme, China |
| Taq Pro Universal SYBR qPCR Master Mix | Vazyme, China |
| Bradford protein assay kit | Beyotime, China |
| Glycine | Biofroxx, Germany |
| Tris base | Biofroxx, Germany |
| SDS | Biofroxx, Germany |
| Protein loading buffer | NCM Biotech, China |
| Protein molecular weight marker | Thermofisher, USA |
| PVDF membrane | Millipore, USA |
| 5× Rapid semi-dry transfer buffer | DoWin, China |
| Ckb antibody | Santa Cruz Biotechnology, USA |
| RUNX2 antibody | Proteintech, China |
| ALP antibody | Proteintech, China |
| OPN antibody | Proteintech, China |
| β-actin antibody | Servicebio, China |
| DRP1 antibody | Wanleibio, China |
| P-DRP1 Ser616 antibody | Proteintech, China |
| MFN1 antibody | UpingBio, China |
| FIS1 antibody | UpingBio, China |
| Cryab antibody | Proteintech, China |
| TOMM20 antibody | Servicebio, China |
| Goat anti-mouse/rabbit IgG-HRP | Abbkine, USA |
| Fluorescent anti-mouse/rabbit IgG | Abbkine, USA |
| Universal antibody diluent | NCM Biotech, China |
| ECL chemiluminescent substrate | Abbkine, USA |
| MitoSOX™ Red mitochondrial superoxide indicator | Thermofisher, USA |
| Mitochondrial membrane potential assay kit | Beyotime, China |
| Antifade mounting medium with DAPI | Beyotime, China |
| DAB detection kit | MXB, China |

**Table S2. The primers used for RT-qPCR**

| **Genes** | **Primers** |
| --- | --- |
| *Ckb*-F | CTCATCGACGACCACTTCCT |
| *Ckb*-R | GTCATTGTGCCAGATACCGC |
| *Alp*-F | AACCTCAAAGGCTTCTTCTT |
| *Alp*-R | ACATGGGGAATGTAGTTCTG |
| *Opn*-F | AGCAAGAAACTCTTCCAAGCAA |
| *Opn*-R | GTGAGATTCGTCAGATTCATCCG |
| *Runx2*-F | AACGATCTGAGATTTGTGGGC |
| *Runx2*-R | CCTGCGTGGGATTTCTTGGTT |
| *β-actin*-F | GTGACGTTGACATCCGTAAAGA |
| *β-actin*-R | GCCGGACTCATCGTACTCC |
| *Fhl1-*F | ATTACTGCGTGGATTGCTAC |
| *Fhl1-*R | CTTCATAGGCCACCACAC |
| *Acta2-*F | ACTACTGCCGAGCGTGAGAT |
| *Acta2-*R | AGGTAGACAGCGAAGCCA |
| *Tsc22d4-*F | GAGAGCCTTATCGTCGAGGT |
| *Tsc22d4-*R | TCTCCACCTTAGCCTTGCC |
| *Cryab-*F | AGCTGCTGCTGAAGGAGTTG |
| *Cryab-*R | CCAGACACCTGTTTCCTTGG |
| *Cnn1-*F | GAAGGTCAATGAGTCAACTCAGAA |
| *Cnn1-*R | CCATACTTGGTAATGGCTTTGA |
| *Myl9-*F | GAGGGCTACGTCCAATGTCT |
| *Myl9-*R | GTCGTGCAGGTCCTCCTTAT |
| *Lama5-*F | AGCCTCTCGACTCACCTCAT |
| *Lama5-*R | GCACCTGTTCTCTCGTGTCA |
| *Susd5-*F | AAAGGGAGTGGTGAACAGCC |
| *Susd5-*R | CCACACGGTCTCTCTTCGTC |

**Table S3. Bulk RNA seq results of mBMSCs transfected with ckb overexpression virus.**

| **Gene name** | **Regulation** | **Q value** | **Log2FC** |
| --- | --- | --- | --- |
| *Fhl1* | UP | 8.04E-31 | 1.336875252 |
| *Acta2* | UP | 8.23E-29 | 1.15125348 |
| *Tsc22d4* | UP | 5.05E-24 | 1.636113347 |
| *Cryab* | UP | 1.58E-23 | 3.578961132 |
| *Cnn1* | UP | 1.49E-21 | 3.519986022 |
| *Myl9* | UP | 6.51E-18 | 1.408897693 |
| *Lama5* | UP | 3.91E-17 | 3.202734081 |
| *Susd5* | UP | 4.17E-17 | 2.111404107 |
| *Tnn* | UP | 1.57E-16 | 1.839369173 |
| *Col8a2* | UP | 1.88E-13 | 1.133177377 |
| *Cacna1c* | UP | 4.51E-12 | 1.633960015 |
| *Flt4* | UP | 4.85E-11 | 5.759275227 |
| *Gprc5c* | UP | 2.73E-10 | 2.536064226 |
| *Igfbp7* | UP | 5.10E-10 | 1.0398145 |
| *Kcnh2* | UP | 6.08E-10 | 1.434398679 |
| *Adgrl1* | UP | 9.62E-10 | 2.534696537 |
| *Mrvi1* | UP | 1.44E-09 | 2.654786504 |
| *Id2* | UP | 9.59E-09 | 1.976363135 |
| *Ankrd1* | UP | 9.74E-09 | 3.685878173 |
| *Tinagl1* | UP | 4.06E-08 | 2.871815039 |
| *Kif5a* | DOWN | 1.95E-13 | -5.216943787 |
| *Galnt16* | DOWN | 2.71E-12 | -1.089076154 |
| *Bnip3* | DOWN | 5.85E-12 | -1.011200447 |
| *Klf4* | DOWN | 8.28E-12 | -1.187972735 |
| *Egr3* | DOWN | 1.07E-09 | -1.758536027 |
| *Slc1a2* | DOWN | 3.19E-09 | -4.900148083 |
| *Adamts8* | DOWN | 8.15E-09 | -1.411239967 |
| *Asns* | DOWN | 7.05E-08 | -1.141281982 |
| *mt-Co3* | DOWN | 9.50E-08 | -1.050701996 |
| *Slc16a3* | DOWN | 6.09E-07 | -1.710446268 |
| *Egr1* | DOWN | 7.14E-07 | -1.170075888 |
| *Atp1a3* | DOWN | 3.55E-06 | -3.81686749 |
| *Adcy1* | DOWN | 5.29E-06 | -5.189279959 |
| *Ptgs2* | DOWN | 7.09E-06 | -1.303566059 |
| *Sparcl1* | DOWN | 1.80E-05 | -2.936939561 |
| *Stc2* | DOWN | 2.04E-05 | -1.283719491 |
| *Cdc6* | DOWN | 3.62E-05 | -2.930828965 |
| *Meg3* | DOWN | 4.69E-05 | -3.209509635 |
| *Mbp* | DOWN | 6.91E-05 | -1.595650269 |
| *Mmp19* | DOWN | 9.73E-05 | -6.179038341 |

**Figure S1. Identification of isolated mBMSCs.**

**(A)** Flow cytometry analysis showing the presence of MSC surface markers (CD29 and CD44) and the absence of hematopoietic lineage markers (CD45 and CD11b). **(B)** ARS staining showing mineralized nodule formation after 14 days of osteogenic induction. **(C)** Oil Red O staining showing lipid droplet accumulation after 21 days of adipogenic induction. Representative results from n = 3 independent experiments are shown.

**Figure S2. Validation of lentiviral transduction efficiency in vivo.**

BMSCs were isolated from mice following two weeks of tail vein injection with lentiviruses. Protein levels of Ckb were analyzed by Western blot. **(A-B)** Ckb expression was significantly knocked down in the Ckbsh group compared to the consh (scrambled shRNA) control group. **(C-D)** Ckb expression was markedly increased in the Ckb OE group compared to the vector control group. All data represent mean ± SD. n=3. Statistical significance: *p<0.05, **p<0.01, ***p<0.001, ****p<0.0001.

**Figure S3. Cellular localization of Ckb in tooth extraction socket tissues.**

Immunofluorescence co-localization of Ckb (red) with **(A)** Sca-1 (mesenchymal stem cell marker, green), **(B)** F4/80 (macrophage marker, green), and **(C)** CD31 (endothelial cell marker, green) in murine extraction sockets at 7 days post-extraction. Representative images from n = 3 mice per group are shown.

**Figure S4. Ckb and Cryab do not affect Erk, Cdk1, or Drp1 Ser637 phosphorylation.**

Western blot analysis and quantitative analysis of p-Erk, Erk, p-Cdk1, Cdk1 and p-Drp1 Ser637 in BMSCs following **(A)** Ckb knockdown, **(B)** Ckb overexpression, and **(C)** Cryab overexpression. All data are presented as mean ± SD, n = 3. ns indicates not significant (p > 0.05).
